# Rewiring Lipid Metabolism: PINK1 as a Central Regulator of Mitochondrial Homeostasis in Parkinson’s Disease

**DOI:** 10.3390/ijms27136073

**Published:** 2026-07-07

**Authors:** Ana Beatriz Ramos, Vanessa Alexandra Morais

**Affiliations:** 1 Faculdade de Medicina (FMUL), Universidade de Lisboa, 1649-028 Lisbon, Portugal; bramos@medicina.ulisboa.pt; 2 Gulbenkian Institute for Molecular Medicine (GiMM), 1649-028 Lisbon, Portugal

**Keywords:** Parkinson’s disease, mitochondria, PINK1, lipids, metabolism

## Abstract

Increasing evidence highlights a tight interplay between lipid metabolism and mitochondrial homeostasis in neurons, with disruptions in either pathway amplifying cellular vulnerability. PTEN-induced kinase 1 (PINK1), a familial Parkinson’s disease (PD)-related gene and a key regulator of mitochondrial quality control and homeostasis, emerges at the intersections of lipid metabolic pathways, influencing membrane composition, fatty acid utilization, and neuronal energy balance. Within this review, we discuss the role of mitochondria as hubs for lipid metabolism, the mechanisms and functional consequences of neuronal lipid handling, and the complex bidirectional relationship between lipid dysregulation and PD pathology. Special focus is given to lipid–mitochondria crosstalk and how PINK1 orchestrates this interface to maintain neuronal homeostasis. Finally, we consider therapeutic perspectives that target lipid and mitochondrial pathways, highlighting strategies to restore cellular function and PD pathology.

## 1. Introduction

Parkinson’s disease (PD) is a progressive neurodegenerative disorder characterized by selective dopaminergic neuronal loss and intracellular buildup of protein aggregates, most notably α-synuclein [1]. While the molecular cornerstones of PD have traditionally centered on protein misfolding, mitochondrial dysfunction, and impaired proteostasis, an increasing body of evidence highlights lipid metabolism as a critical and previously underappreciated contributor to disease pathogenesis [2]. Lipids are not only essential structural components of cellular membranes but also serve as signaling molecules and metabolic substrates, playing key roles in energy homeostasis, vesicular trafficking, and organelle communication. Disruptions in lipid composition and distribution have been linked to altered membrane dynamics, defective synaptic transmission, and enhanced vulnerability to oxidative stress—all hallmarks of neurodegeneration in PD [3,4]. Genetic and lipidomic studies have identified alterations in key lipid classes, including phospholipids, sphingolipids, and cholesterol derivatives, further underscoring the systemic nature of metabolic dysregulation in PD.

Among the genetic factors implicated in familial forms of PD, PTEN-induced kinase 1 (PINK1) has emerged as a central regulator of mitochondrial quality control. PINK1 is best known for its role in initiating mitophagy through the recruitment and activation of Parkin, thereby ensuring the selective removal of damaged mitochondria from cells [5]. Under basal conditions, PINK1 is responsible for other essential mitochondrial functions, most notably the control of the Electron Transport Chain (ETC) activity and energy production [6]. Accumulating evidence indicates that PINK1 also plays a broader role in modulating mitochondrial homeostasis, particularly in lipid metabolism and membrane integrity. PINK1 has been shown to influence lipid trafficking, regulate enzymes involved in fatty acid β-oxidation (FAO), and modulate the composition of mitochondrial membranes [7,8,9,10], suggesting that it acts as a key node integrating metabolic and quality control pathways, both implicated in the disease.

In this review, we examine the growing evidence that positions alterations in lipid metabolism as a central player in PD pathophysiology and explore how PINK1 contributes to the rewiring of these pathways. We discuss the molecular mechanisms by which lipid alterations impact mitochondrial function and neuronal viability and highlight the multifaceted role of PINK1 in coordinating lipid homeostasis, organelle crosstalk, and mitochondrial quality control. By integrating insights from both experimental disease models and PD patients, this review aims to provide a comprehensive framework for understanding how metabolic dysfunction converges on mitochondrial pathways in PD and to identify potential therapeutic opportunities targeting lipid metabolism and PINK1 signaling.

## 2. PINK1 Dysfunction and Mitochondrial Impairment in Parkinson’s Disease

PD is the second-most common neurodegenerative disease worldwide, being characterized by an age-dependent progressive loss of neurons, particularly of the dopaminergic neurons in the substantia nigra pars compacta, and by the intracellular accumulation of Lewy bodies. This selective degeneration leads to motor symptoms such as bradykinesia, tremors and limb rigidity, although non-motor symptoms are also prevalent and normally precede the motor manifestations [11].

PD is an etiological complex disorder, in which both environmental and hereditary variables play a role, and although most cases are sporadic, different risk factors have been identified and linked to the disease [12,13,14]. PD is considered a predominately age-dependent disorder, resulting from an interaction between multiple genetic and environmental risk factors. Despite this, some PD cases are caused by rare familial genetic mutations. Approximately 10% of PD cases have a genetic cause that presents as a Mendelian form with autosomal dominant or recessive inheritance. Mutations in genes such as PARK2 (Parkin), LRRK2/PARK8 (Leucine rich repeat kinase 2), SNCA-PARK1/PARK4 (α-synuclein), PINK1/PARK6 (PTEN-induced putative kinase 1), PARK7 (DJ1), UCH-L1 (Ubiquitin C-terminal hydrolase like 1) and ATP13A2 (ATPase13A2) have been identified and are among the most prevalent mutations found in young-onset PD [15,16]. Although pathological mutations in these genes affect several cellular functions, they appear to directly or indirectly impact mitochondria. Alterations in the normal mitochondrial function therefore represent a common mechanistic link between sporadic and genetic PD forms and is widely regarded as a defining pathological feature of the disorder [17].

### 2.1. Molecular Mechanisms of PINK1 Function in Mitochondrial Homeostasis

Among the PD-associated genes, mutations in the PINK1 gene (encoding for the PTEN-induced putative kinase 1 protein) further strengthen the link between PD and mitochondria dysfunction.

PINK1 is a mitochondrial targeted serine/threonine kinase protein that plays an essential role in maintaining mitochondria quality control and overall homeostasis. Structurally, PINK1 is composed of a C-terminal, a highly conserved kinase domain, a transmembrane domain, and an N-terminal mitochondrial targeting sequence (MTS), which primarily localizes PINK1 to the mitochondria [18]. The function of PINK1 in this organelle has been linked to alterations in the mitochondrial membrane potential, which acts as a switch for the PINK1-mediated activities. Evidence suggests that when mitochondria are damaged, PINK1 is recruited to the outer mitochondrial membrane (OMM) [19], where it will be activated, and recruit and phosphorylate the E3-ubiquitin ligase Parkin at the Serine 65 residue [20,21]. PINK1 activity is also critical for the phosphorylation of ubiquitin in this same residue. Both PINK1 phosphorylation of Parkin and formation of phospho-ubiquitin contribute to Parkin activation and ubiquitination of several mitochondrial proteins [22]. This leads to the recruitment of autophagic adaptor proteins, including p62, NBR1, NDP52 and OPTN [23,24,25]. Ultimately, these adaptors bind to Microtubule-associated protein 1 light chain 3 (LC3) on autophagophore membranes, supporting the engulfment of depolarized mitochondria into autophagosomes that, when complete, can be degraded by lysosomes [26]. This process, commonly termed mitophagy, is critical for maintaining cellular homeostasis, energy metabolism, and quality control, preventing accumulation of toxic molecules including reactive oxygen species (ROS) and cell death.

Under physiological conditions, when the mitochondrial membrane potential is normal, PINK1 protein levels are tightly regulated through continuous translocation to the inner mitochondrial membrane (IMM) via the mitochondrial protein-transporting ATPase translocase (TOM/TIM complex), which is followed by proteolytic processing and degradation [27]. PINK1 is a short-lived protein with a very well-described import/cleavage processing, and it is well established that PINK1 can phosphorylate different substrates once inside the mitochondria.

Among the first reported PINK1 phosphorylation substrates is the tumor necrosis factor (TNF) receptor-associated protein 1 (TRAP1), a molecular chaperone also known as heat shock protein 75 (Hsp75). It has been shown that PINK1 associates with TRAP1 in the mitochondria and phosphorylates this protein, protecting against oxidative stress and promoting cell survival [28]. Studies have also demonstrated that upregulation of TRAP1 in PINK1 mutant flies is able to restore mitochondrial defects [29,30], further strengthening this link. The high-temperature requirement A2 (HtrA2) is a mitochondrial serine protease that has also been found to be phosphorylated in a PINK1-dependent way following activation of the Mitogen-activated protein kinase (MAPK) signaling cascade, a process that regulates apoptosis and is initiated upon environmental stress or inflammation. A study has demonstrated that, after rotenone or 6-hydroxy dopamine (6-OHDA)-induced stress, PINK1 phosphorylates HtrA2 and modulates its proteolytic activity, protecting cells against mitochondrial stress [31]. Very interestingly, PINK1 has been identified as an important regulator of the ETC reduced Nicotinamide Adenine Dinucleotide (NADH):ubiquinone oxidoreductase (Complex I), via the phosphorylation of its subunit NDUFA10 [32]. Several studies have reported defects in Complex I activity in PINK1 mutants [6,33], which is in line with the observed defects also found in PD patients and models [34,35]. Also, as several other reports have highlighted alterations in different cellular pathways due to PINK1 defects, one can expect that additional PINK1 substrates have yet to be identified.

The reports on these PINK1 phosphorylation substrates widen the scope of PINK1’s function in cells aside from its typical assigned role in mitophagy. Moreover, they highlight the importance of PINK1’s function in essential cellular processes including regulation of energy metabolism, apoptosis, and molecular signaling, supporting the critical role of mitochondria in cells, particularly in the brain.

### 2.2. Mitochondria as Master Regulators of Neuronal Homeostasis and Activity

Mitochondria are highly functional organelles that play several important roles in cells, and whose integrity is of paramount importance for neuronal function and survival [36,37]. Bioenergetically, these organelles provide a significant part of the neuron’s energy, in the form of adenosine triphosphate (ATP), to regulate the normal cellular processes, including synaptic transmission, axon maintenance and growth, and apoptosis [38,39,40]. Due to their high energetic needs, neurons are particularly reliant on functional mitochondria to sustain their activity and survive. Mitochondria also play an important role in calcium buffering, maintaining its homeostasis, and regulating its transport, processes that are crucial for normal cellular function [41]. In the case of neurons, calcium signaling is also especially important to regulate several aspects of neurotransmitter release and synaptic function [42]. Mitochondria also play a critical role in the brain’s metabolic signaling, especially through the tricarboxylic (TCA) cycle. After shuttling to the cytosol, intermediates from this cycle exert metabolic influences on cell fate and function, namely through the regulation of genes and the epigenome, immune responses, and stem cell activation [43].

Due to their complex morphology, neuronal cells also rely on mitochondria to sustain their more distal activities. Mitochondria are highly dynamic and travel along microtubules, in both an anterograde and retrograde way, via specialized motor proteins. Such transport is required to ensure proper mitochondrial distribution and turnover in the dendritic and axonal compartments [44,45]. During neuronal development, mitochondrial positioning at growing axons is essential for axonal architecture [46]. Local protein synthesis within axonal and somatodendritic compartments is a critical component of neuronal homeostasis and requires mitochondrial energy supply to sustain local translation, especially in the context of synaptic plasticity [37]. Finally, many nuclear-encoded mitochondrial proteins have been identified in both dendritic and axonal compartments, both at transcriptomic and proteomic levels [47,48], and local mitochondrial protein synthesis has been revealed to maintain mitochondrial morphology and membrane potential [49].

Mitochondrial function is also dictated by its morphology. In the case of neurons, mitochondrial fusion and fission events that occur in dendrites and axons of different neuronal populations are responsible for controlling mitochondrial shape and size [50,51]. Mitochondrial fission and fusion are regulated by multiple proteins, including Dynamin-1-like protein (DRP1), Mitochondrial fission 1 protein (FIS1), Mitochondrial fission factor (MFF), Mitofusin-1 (MFN1), Mitofusin-2 (MFN2), and Dynamin-like GTPase (OPA1), which together maintain the proper balance between these processes and thereby support mitochondrial homeostasis [52,53,54]. The differences in mitochondrial morphology across different neuronal compartments are essential to sustain normal mitochondria functions. Decreased mitochondrial fission has been shown to impact calcium buffering via increased ion entry following synaptic activity, leading to defects in synaptic vesicle release and decreased axonal branching. Interestingly, this had no effect on ATP levels or mitochondrial membrane potential [55,56]. Similar observations have also been made regarding dendritic development and maintenance, as well as spine remodeling [57,58]. Bioenergetically, the positioning of mitochondria at synaptic terminals has also been implicated in the metabolism of fatty acids (FAs), with local mitochondria having a higher capacity to metabolize these lipids compared with the non-synaptic pool [59].

Considering these features, it is not surprising that altered mitochondrial function and homeostasis are hallmarks of neurodegeneration and key contributors to the pathophysiology of PD. Importantly, the current view of PD-related mitochondrial impairment has expanded beyond classical defects in quality-control pathways to include broader alterations in cellular organization, such as disruptions in lipid metabolism, membrane composition and organelle dynamics. These interconnected processes are likely to play a central role in determining neuronal vulnerability in PD. In this context, accumulating evidence highlighting disturbances in lipid metabolism in PD further supports a framework in which lipid dysregulation contributes significantly to disease progression.

## 3. Mitochondria as Hubs for Lipid Metabolism and Homeostasis

Mitochondria are double-membrane organelles in the cell, composed of two layers (OMM and IMM), the intermembrane space (IMS) and the matrix, which is the innermost space of mitochondria. The IMM forms invaginations towards the matrix called cristae, which significantly increase the area of this membrane and provide more space for the main function of mitochondria in cells—the oxidative phosphorylation (OXPHOS).

Aside from being essential energy-providers for cells, mitochondria are also important for the overall cellular physiology, controlling cell apoptosis, regulating ROS generation, calcium and ATP levels, and participating in signaling cascades that ultimately confer cells with protection and ensure its proper activities. Among the many functions played by these organelles, mitochondria play a key role in lipid metabolism and homeostasis, processes that are differently regulated depending on the type of tissue. Despite these tissue differences, it remains uncontested that alterations in lipid metabolism are associated with multiple neurodegenerative diseases and metabolic disorders [60,61].

Lipids are a fundamental class of organic biomolecules implicated in a wide range of biological mechanisms related to their structural diversity. They are crucial elements of cellular membranes and primary energy stores, playing important roles in the maintenance of cellular integrity and function. In general, lipids can be classified into five main categories, which include FAs, triglycerides, phospholipids, sterol lipids and sphingolipids. This class of molecules is highly diverse, reflecting on the importance of their role in the mammalian biological systems [62].

As the main bioenergetic hubs of cells, mitochondria are equipped to process lipids, which can be degraded via FAO to produce ATP through OXPHOS, or can be synthesized via fatty acid synthesis (FAS) pathways to provide cells with long-term energy storage, assist in membrane formation, or create signaling molecules. Depending on the tissue, cells may rely on FAs for energetic purposes. FAs are important nutrients for cells, and their storage as triglycerides in adipose tissue enables high tolerance to prolonged periods of starvation or fasting, as well as in other metabolically demanding conditions, such as during exercise. Cells can obtain FAs through two primary mechanisms: direct uptake from the diet (exogenous source) and de novo biosynthesis (endogenous source). The process by which FAs can be used for energy is called FA β-oxidation, a mechanism that takes place in the mitochondrial matrix. For this process to occur, FAs first need to enter the cell by crossing the plasma membrane [63]. This transport depends on the FA length, with long-chain FAs (LCFAs) requiring the aid of specific proteins that reside in the cell membrane, whereas medium-chain FAs (MCFAs) and short-chain FAs (SCFAs) can cross by simple diffusion. Many membrane-associated FA transporters assist in the cellular entry of LCFAs, including the peripheral membrane Fatty-acid-binding protein (FABP_pm)_ which pairs up with the cytoplasmic FABP_c_, the transmembrane transporter CD36, and the FA transporter protein (FATP) [64].

Before entering the mitochondria, FAs first need to be oxidized through the conversion to fatty acyl-CoA by acyl-CoA synthetase enzymes in the cytosol [65]. After this reaction is completed, these substrates are transported across the mitochondrial membranes, a process that is tackled by the carnitine palmitoyltransferases (CPTs) [66]. In the OMM, the CPT1 protein converts the fatty acyl-CoAs into fatty acylcarnitines, mediating their transport into the intramembrane space. This is considered the rate-limiting step for the FAO as it controls whether fatty acyl-CoAs will be used as fuel for ATP production depending on the energy requirements of the cell. For this regulation, the CPT1 activity is tightly controlled by the malonyl-CoA metabolite. After crossing the OMM, fatty acylcarnitines are translocated into the matrix through the IMM by the carnitine–acylcarnitine translocase (CACT), in exchange for free carnitine. On the matrix, CPT2 converts the substrate back to fatty acyl-CoA, preparing it to enter the FAO pathway [67].

Within the mitochondrial matrix, acyl-CoAs are degraded via a cyclic mechanism consisting of four enzymatic reactions catalyzed by acyl-CoA dehydrogenase (ACAD), enoyl-CoA hydratase, hydroxyacyl-CoA and ketoacyl-CoA thiolase enzymes [65]. Each cycle generates one molecule of reduced Flavin adenine dinucleotide (FADH2), one molecule of NADH, one molecule of acetyl-CoA and a fatty acyl molecule with two less carbon atoms. This process is repeated until the entire carbon backbone is broken down. In the case of an odd-numbered carbon chain, propionyl-CoA is produced as the final product of the reaction. The FADH2 and NADH transfer their free electrons to the respiratory chain to produce ATP, while acetyl-CoA directly fuels the TCA cycle and the ketogenesis pathway. The ACAD enzymes use enzyme-bound FAD as their intermediary electron acceptor, and the FADH2 molecules that result from the ACAD enzymatic reaction are oxidized in the mitochondrial matrix by the electron transfer flavoprotein (ETF), comprising ETF subunit A and B (ETFA and ETFB), which then transfers the electrons to the ETF dehydrogenase, culminating in the donation to the coenzyme Q and direct feeding to the respiratory chain [68].

Apart from being important energetic substrates, lipids are also fundamental building blocks in all cells, playing a plethora of other essential roles. These biomolecules are key components of the plasma membrane, of other cellular compartments, including the endoplasmic reticulum (ER), the Golgi apparatus, and the nucleus, and of trafficking vesicles, including lysosomes and endosomes. There are significant differences in the lipid composition across organelles and cell types, which indicates that different lipids are essential for distinct functions [69]. Mitochondria also have a unique membrane lipid composition, mainly comprising phosphatidylcholine (PC), phosphatidylethanolamine (PE), and its signature phospholipid cardiolipin (CL). Other complex lipids are also found in this organelle at lower concentrations, including cholesterol, sphingolipids, ceramide and others. The lipid composition and function of the mitochondrial membrane have been extensively reviewed in [70]. Lipids can also act as first and second messengers in signal transduction and other molecular recognition processes. All cells use chemical messengers to communicate with each other and to be able to respond to environmental cues. Furthermore, the different intracellular compartments and organelles must also interact via signal transduction. The cellular membrane and its constituent lipids are therefore indispensable players in these signaling events, acting as docking sites for cytoplasmic signaling proteins or to assist in the generation of ligands or substrates for other signaling pathways. Non-membrane lipids can also function as ligands, and post-translational lipid modifications can provide ways for proteins to associate with membranes [71].

Under conditions of high oxidative stress, when mitochondria are dysfunctional, lipids can become targets of lipid peroxidation, a biochemical process where oxidants, including free radicals or ROS, inflict direct damage on these molecules. Although the main targets are the lipids containing carbon–carbon double bonds, including polyunsaturated FAs (PUFAs), glycolipids, phospholipids and cholesterol have also been found to be modified by this process. Enzymes such as lipoxygenases, cyclooxygenases and cytochrome P450 can also oxidize lipids [72]. Depending on the specific cellular circumstances and repair capacities of cells, lipid peroxidation can either activate cell survival mechanisms or induce cell death.

To conclude, mitochondria play a central role in lipid metabolism by coordinating FAO and the generation of lipid-derived metabolites that contribute to cellular energy production, membrane biogenesis, and signaling pathways. Through these processes, mitochondria help maintain lipid homeostasis and support essential cellular functions across different tissues. In the brain, however, lipid metabolism is particularly complex and tightly regulated, reflecting the high energetic demands and specialized architecture of neural cells. Neurons rely on precise lipid composition to sustain membrane integrity, synaptic transmission, and intracellular signaling, while mitochondrial activity is critical for meeting their metabolic needs. Consequently, disturbances in mitochondrial function can profoundly affect lipid homeostasis in the brain, potentially compromising neuronal viability and function. Hence, understanding how lipid metabolism is regulated in neurons is therefore essential to elucidate the mechanisms that sustain neuronal physiology and to uncover how its dysregulation may contribute to neurodegenerative processes.

## 4. Neuronal Lipid Metabolism: Mechanisms and Functional Implications

**Figure 1:**
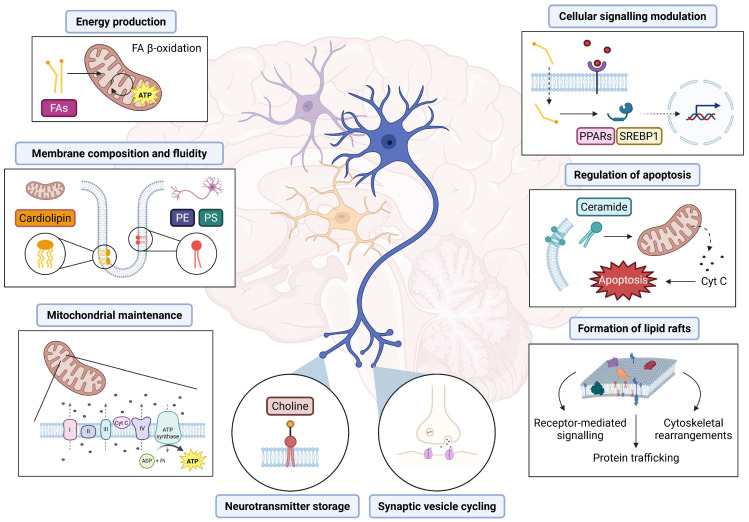
Lipid homeostasis is essential for sustaining neuronal health and function. Fatty acids (FAs) are used as fuels by neuronal mitochondria by undergoing successive β-oxidation cycles, generating energy in the form of adenosine triphosphate (ATP) and other important metabolic intermediaries. Lipids such as cardiolipin, phosphatidylethanolamine (PE) and phosphatidylserine (PS) ensure a proper membrane composition and conformation, essential for sustaining membrane integrity and neuronal compartmentalization. Functionally, these membranes can store precursors for neurotransmitter synthesis and modulate vesicle formation, vital processes for effective synaptic transmission. In neuronal mitochondria, multiple lipid species contribute to maintaining the electron transport chain function and a balanced membrane potential. Lipids are important bioactive molecules that regulate multiple cellular signaling pathways, including peroxisome proliferator response receptors (PPARs) and sterol regulatory element-binding protein 1 (SREBP1)-mediated transcription. These molecules can also trigger other cellular responses, including apoptosis, via ceramide-mediated cytochrome C (Cyt C) release, resulting in the activation of neuroinflammation cascades. Finally, lipid rafts, which are mainly composed of sphingolipids and cholesterol, are important constituents of cellular membranes, and act as hubs for protein organization/transport, signaling and cellular structural remodeling. I: Complex I; II: Complex II; III: Complex III; IV: Complex IV; Cyt C: Cytochrome C; ADP: Adenosine diphosphate; Pi: Inorganic phosphate; ATP: Adenosine triphosphate; FAs: Fatty acids; PE: phosphatidylethanolamine; PS: phosphatidylserine; PPARs: peroxisome proliferator response receptors; SREBP1: sterol regulatory element-binding protein 1. Created in BioRender. Ramos, A. B.; Morais V.A. (2026) https://BioRender.com/siix6wn (accessed on 7 April 2026).

Even though the adipose tissue serves as the main lipid reservoir of the body, the brain contains the second-highest concentration of lipids (approximately 60% of its dry weight) [73]. All classes of lipids play an important role in the brain, being particularly essential for the structural complexity and functional relevance of neurons. Lipids form the structural foundation of membranes and create insulating myelin sheaths for a quick signal transduction. They also act as vital signaling molecules for synaptic plasticity and confer stability to different structures. Finally, they can provide energy to perform all the required neuronal activities including neurotransmitter release and recycling. Due to all these factors, it comes to no surprise that lipids are key to neuronal development, plasticity and function (Figure 1).

The homeostasis of lipids in neurons is also greatly impacted by glial cells in the brain. Metabolic coordination between neurons and astrocytes is essential for normal brain function, with astrocytes assimilating and processing excess free FAs via FAO during high-neuronal activity periods. This provides a detoxification mechanism that sustains both neuronal and astrocytic homeostasis [74]. Neurons critically rely on oligodendrocytes to form and maintain their myelin sheaths, which are composed of cholesterol, phospholipids and glycolipids. This process ensures a proper axonal function and directly affects action potential propagation [75]. Finally, lipid metabolism modulates microglia activation and inflammation in the brain, which in turn can induce multiple signaling cascades and result in neuronal inflammation and cell death [76]. Therefore, neurons depend on the overall brain environment to sustain their lipid metabolic functions.

### 4.1. Lipids as Energy Substrates in the Brain

As one of the most energy-demanding organs, the brain requires a near-continuous source of metabolites to sustain its functions. Although there is a general consensus that glucose is the dominant energy substrate satisfying most of the brain’s energy needs, studies have shown that approximately 20% of the total energy requirement of this organ is met through the oxidation of FAs, particularly through astrocytes [77].

The β-oxidation of FAs is a very positive energy-balanced process compared with glycolysis. While the degradation of the long-chain palmitic acid (PA) renders a net gain of 106 ATP molecules, the metabolism of glucose generates at most 36 ATP molecules per glucose molecule [78,79]. Even though the brain would benefit more from using lipids rather than sugars as its main energy fuel, the oxygen requirements for both energetic metabolisms is rather different. While glycolysis is mainly an anaerobic process able to generate energy even when oxygen reserves are low, the FAO is an oxygen-dependent process, consuming a great amount of the brain’s oxygen reserves. Moreover, the β-oxidation pathway releases high amounts of ROS, particularly in the form of superoxide that, when accumulated, is harmful for neurons [80,81]. Dopaminergic neurons are particularly susceptible to this enhanced oxidative stress due to their structural complexity and distinct physiological features, which might render them more vulnerable to bioenergetic burden [82,83].

Evidence indicates that free FAs can modulate mitochondrial oxidative phosphorylation through their uncoupling activity, thereby influencing both respiratory efficiency and reactive oxygen species production in a context-dependent manner [84,85]. Therefore, considering that the brain is a very fragile organ with low oxygen storages, that it is greatly affected by small alterations in its environment [86], glucose becomes a mandatory energetic substrate for normal and sustained brain activity. However, under low-glucose conditions such as fasting or prolonged exercise, lipids can prove to be a more valuable energy source. This occurs via the use of ketone bodies, lipid-derived metabolites produced in the liver during fasting through β-oxidation of FAs mobilized from adipose tissue. Under these conditions, FA-derived acetyl-CoA can be used in the ketogenesis pathway to generate one of the three types of ketone bodies (acetoacetate, D-3-hydroxybutyrate and propanone) [87]. These ketone bodies are then shuttled into the bloodstream to be transferred to the different organs of the body, including the brain [88]. Once in the brain, these particles can be degraded through ketolysis, which will revert ketone bodies back to acetoacetyl-CoA, and consequently to acetyl-CoA, which can enter the TCA cycle to produce ATP. These pathways have been well described to occur in both neurons and glial cells [89,90], highlighting the importance of ketones as brain fuels under specific conditions.

Many studies appear to suggest that the brain can use FAs as fuel for energy production. A study conducted in CPT2 knockout (KO) mice showed increased transcript levels of β-oxidation-related enzymes and accumulation of long-chain acylcarnitines in the animals’ cortices and hippocampi, respectively, demonstrating the existence of FA metabolic activity within the mammalian brain under normal conditions, particularly associated with astrocytic cells [91]. Evidence of lipid droplet (LD) dynamics in the brain includes their presence in astrocytes co-cultured with neurons, where they participate in FA storage and mobilization, contributing to protection against FA-induced toxicity [74]. LDs are dynamic organelles involved in lipid storage and cellular stress responses, and in neurons, their accumulation has been primarily associated with conditions of metabolic stress and neurodegeneration, including regulation of autophagy and cellular homeostasis [92,93,94,95]. To provide a tight and timely regulation of these structures, LDs are in close contact with mitochondria and their interactions tightly regulate LD dynamics [96]. Even though the relevance of mitochondrial FAO in neurons has remained controversial throughout the years, with the current dogma still claiming a preferential use of glucose as the main fuel, more evidences have been challenging this hypothesis and showing that neurons, apart from other brain cell populations, can use lipids to support their functions under basal conditions, highlighting this process as part of the brain metabolism. In Drosophila flies, mitochondrial FAO plays a critical role in ATP production in neurons from the mushroom body, being essential for cognitive function and memory formation [97]. Genetic ablation of the triglyceride and phospholipid lipase DDHD2 in cultured mouse hippocampal neurons hinders the mitochondrial bioenergetic function and impairs energy production via a deficient release of saturated free FAs [98]. This occurs independently of glucose metabolism, and a combination of different activated FAs was able to restore deficiencies in membrane trafficking, protein homeostasis and synaptic function.

In line with this, a study revealed that blocking DDHD2 in neuronal synapses induces accumulation of LDs in nerve terminals, and that the activity of this lipase is modulated electrically to regulate the flux of FAs from LDs to mitochondria, providing fuel for energy production [99]. This suggests that a regular and steady FA flux in neurons is key to sustaining synaptic function. The endocytic and intracellular receptor Sortilin 1 (SORT1) has been shown to facilitate neuronal uptake of apolipoprotein E (ApoE), thereby regulating lipid transport in the brain [100]. Studies performed on humanized mouse strains and induced pluripotent stem cells revealed that SORT1 mediates the uptake of polyunsaturated FAs bound to ApoE isoform 3 (ApoE3), and that these lipids are converted into ligands for the peroxisome proliferator response receptor (PPAR) α transcription factor, activating transcription profiles that enable neurons to use LCFAs as alternative metabolic fuels [101]. This pathway supports neuronal energy homeostasis under both normal and glucose-restricted conditions, and is compromised in Alzheimer’s disease (AD), since the disease risk isoform ApoE4 disrupts SORT1 endocytic activity. All this evidence supports the hypothesis that lipids, in the form of FAs, can be used by the brain as a primary fuel for energy production rather than exclusively under particularly demanding conditions.

### 4.2. To Energy and Beyond: The Roles of Lipids Outside FA β-Oxidation

Aside from their role as energy molecules, lipids are critical components of the cellular structural machinery. Although they mainly integrate the plasma membrane, they are constituents of all membranes of the cell, rendering them quite crucial due to the compartmentalization of the several neuronal signaling processes [102]. Phospholipids form the main structural unit denominated the phospholipid bilayer and are formed by different subclasses, each one playing a particular role and conferring functional diversity to the membranes. In cholinergic neurons, choline is a major component of the membrane lipid PC, which simultaneously serves as a storage pool for the synthesis of the neurotransmitter acetylcholine [103]. Therefore, membranes containing this phospholipid are crucial for normal brain function and proper synaptic activity. PE is enriched in the PUFA arachidonic acid, a fundamental precursor of multiple neuromodulatory signaling molecules, including prostaglandins and anandamides. Moreover, PE is thought to enable vesicular budding and membrane fusion by conferring higher curvatures and fluidity to membranes [104,105]. The negatively charged phospholipid phosphatidylserine (PS) is thought to function as a regulator of several proteins and acts as a buffer for some bioactive FAs including docosahexaenoic acid, essential for brain maintenance and function [106,107]. Phosphatidylglycerol (PG) is found in low concentrations in eukaryotic mitochondrial membranes [108] and acts as a precursor of CL, a major component of mitochondrial membranes [109]. CL is responsible for maintaining the membrane potential in the IMM and helps sustain the activity of the ETC complexes, being indispensable for neuronal function and survival [110,111].

Sphingolipids are a different class of structural lipids, that together with cholesterol, constitute lipid rafts. These rafts are small, and dynamic platforms within cellular membranes that can act as organizing centers for proteins and have been implicated in different cellular pathways including signal transduction and protein trafficking [112]. Such functions are critical in the brain, particularly for neuronal synapse development and maturation, and when compromised, they can lead to synaptic loss [113,114,115].

Lipids are also important bioactive molecules that regulate multiple cellular signaling processes. FAs, particularly PUFAs, have been shown to modulate different neuronal signaling processes including neurogenesis, brain vesicular formation and transport, glucose homeostasis, and endocannabinoid regulation, among others [116,117,118,119]. The role of PUFAs in the brain is mediated by the FA-activated receptors PPARs (particularly PPARδ and PPARβ), which are involved in the regulation of FA metabolism and inflammatory responses [120,121,122,123]. These molecules also regulate the function of the sterol regulatory element-binding protein 1 (SREBP1), a master transcription factor of genes involved in lipid and cholesterol synthesis, that has an important role in controlling fat storage and metabolism [124].

Despite playing a role in the integration of cellular membranes, phosphatidylinositol (PI) acts as a crucial precursor of the phosphoinositides (PIPs), signaling molecules that regulate cell growth, membrane trafficking, cytoskeleton organization and signal transduction pathways. When phosphorylated, PI activates phospholipase C, producing inositol triphosphate (IP_3_) and diacylglycerol [125,126]. IP_3_ is shuttled rapidly to the cytosol where it promotes calcium release [127], therefore prompting inter-neuronal communication through vesicular-mediated signaling.

Ceramides are precursors of all complex sphingolipids. Functionally, they are powerful regulators of brain homeostasis, being present at high levels in neural cellular membranes [128]. The intracellular levels of ceramide are greatly regulated in these cells, with alterations of the sphingolipid–ceramide ratios contributing to the development of several age-related, neurological and neuroinflammatory disorders. Ceramides are essential for the proper function of the brain [129,130], participating in the regulation of apoptosis, cellular function and overall metabolism [131].

In conclusion, lipids play a critical role in providing energy, shaping cellular membranes and modulating biochemical cascades in neurons, both through the intricacies of individual lipid classes and through complex associations of several species. These processes need to be tightly controlled to unsure the maintenance of neuronal integrity and survival. Compromising such pathways could therefore result in significant alterations in normal brain function, which could lead to neurodegeneration and ultimately disease. In line with this hypothesis, evidence has strongly linked alterations in lipid homeostasis to several neurodegenerative disorders, including PD, suggesting a mechanistic role for this class of biomolecules in disease pathophysiology [132].

## 5. Lipid Dysregulation in PD: Untangling Cause and Consequence

**Figure 2:**
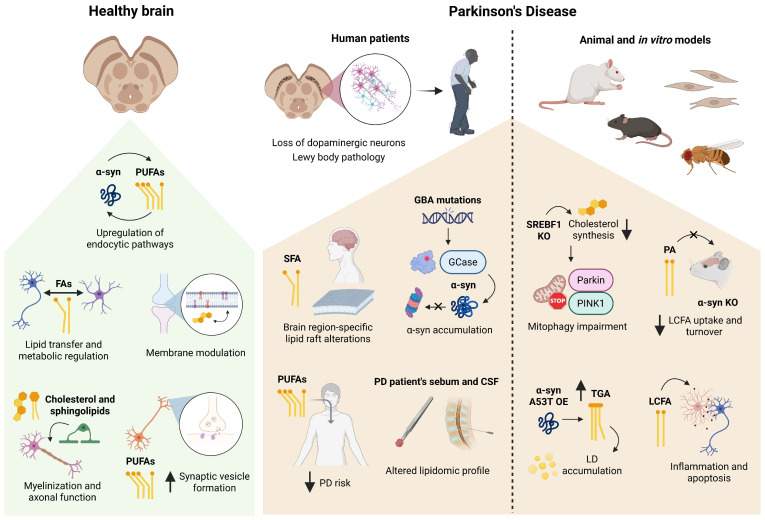
Importance of lipid homeostasis in healthy brain and PD-related lipid metabolism modifications. In the healthy brain, balanced FAs (particularly SFA and PUFAs) metabolism are essential for proper neuronal function, including energy production. This process is highly regulated by astrocytes that transport these molecules to neurons and further act as central “recycling” platforms for toxic lipid accumulation via lipid droplet storage. Other lipid species, including sphingolipid–cholesterol interactions, support membrane organization, synaptic vesicle dynamics and efficient synaptic transmission. In PD, post-mortem brain tissue reveals significant lipidome alterations, as well as different lipid raft compositions in specific brain areas. Dietary PUFA intake is linked with a lower risk of PD development. Mutations in the GBA gene and consequent impairment in GCase activity are suggested to promote α-synuclein accumulation and disrupt lipid homeostasis. Studies on PD models further strengthen this link, showing defective LCFA (in the form of PA) uptake to the brain of α-synuclein mutant rats, as well as LCFA treatment of primary neural cultures resulting in the release of pro-inflammatory factors and contributing to cell death. Overexpression of mutant α-synuclein causes increased TAG synthesis and lipid droplet accumulation, and impaired cholesterol synthesis derived from SREBF1 KO disrupts PINK1/Parkin-dependent mitophagy, exacerbating mitochondrial dysfunction. These converging lipid alterations drive neuroinflammation, synaptic dysfunction, and ultimately neuronal loss. α-syn: α-synuclein; FAs: Fatty acids; PUFAs: Polyunsaturated FAs; SFA: Saturated FAs; LCFA: Long-chain FA; PA: Palmitic acid; TGA: Triacylglycerol; LD: Lipid droplets; GBA: Glucosylceramidase-beta; SREBF1: Sterol Regulatory Element-Binding Transcription Factor 1; GCase: Glucocerebrosidase; PINK1: PTEN-induced putative kinase 1; PD: Parkinson’s disease; CSF: Cerebrospinal fluid; KO: Knockout; OE: Overexpression. Arrow notation: Bold arrows indicate increased and decreased processes, respectively. Thin arrows indicate the direction of biological processes or conceptual/functional relationships between components and should not be interpreted as changes in activity.Created in BioRender. Ramos, A. B.; Morais V.A. (2026) https://BioRender.com/wxcgv9x (accessed on 16 April 2026).

More recently, the importance of lipid homeostasis has been highlighted in the context of neurodegenerative disorders, including PD. Modifications in these pathways are at the base of key pathological mechanisms, including α-synuclein aggregation, organelle dysfunction, oxidative stress and neuroinflammation, which are significantly affected in PD patients and models (Figure 2). However, such lipid alterations may also be evoked by pathological cascades triggered during disease mechanisms, among which the most prominent is mitochondrial dysfunction.

Biochemical and lipidomic studies have uncovered differences in multiple lipid classes, particularly acyl-CoA FAs, in the context of PD. Increased levels of saturated FAs (SFAs) (mainly with lengths of 16:0 and 18:0) have been found in lipid rafts from the frontal cortex of PD patients compared with healthy controls [133]. These changes were not found in other regions of the brain, including the temporal cortex [134], pointing towards region-specific alterations. Post-mortem cerebrospinal fluid (CSF) lipidomic analysis of PD patients revealed imbalances in multiple lipid species, consistent with an association between the disease and lipid homeostasis [135]. Modifications in several lipid metabolites, including sphingolipids and FAs, were also detected in sebum of PD patients following metabolomic profiling [136]. Furthermore, although current evidence does not suggest a link between their intake and PD risk in humans, SFA consumption has been inversely correlated with the development of PD, and dietary FA consumption appears to modulate the association between PD risk and pesticide exposure [137]. In PRKN mutant flies, SFA 18:0 dietary supplementation was found to boost mitochondrial function via activation of the signaling pathways mediated by the transferrin receptor 1 (TRF1) [138]. This impacted mitochondrial fusion, counteracting the PD-induced mitochondrial and behavioral defects [139,140]. In mice subjected to 1-methyl-4-phenyl-1,2,3,6-tetrahydropyridine (MPTP) treatment, intra-gastric gavage of SFA 18:0 significantly attenuated dopaminergic neuron transmission deficits [141]. Treatment of primary hypothalamic neurons and astrocytes with the LCFA PA induces the release of pro-inflammatory cytokines and downregulates peroxisome proliferator-activated receptor gamma coactivator 1-alpha (PGC-1alpha) and estrogen receptor alpha (ER-alpha) expression, triggering inflammation and ultimately resulting in apoptosis [142,143]. This inflammatory pattern has also been found in PD patients [144]. Lastly, the omega-3 PUFA appears to have neuroprotective roles in PD animal models, with studies showing that supplementation with this lipid derivative rescues the behavioral and neurochemical disturbances observed in MPTP-treated mice and 6-OHDA-treated rats [145,146]. Other alterations in different lipid classes have been extensively reviewed in [147].

Evidence has also revealed that the PD-related protein α-synuclein seems to play an important role in the modulation of lipid metabolism. This occurs because physiologically, both α-synuclein and PUFAs contribute to endocytic pathways that support synaptic vesicle recycling during neuronal activity [148]. Furthermore, α-synuclein and PUFAs coordinate with each other functionally, with the FA interacting with the protein’s N-terminal and enhancing its oligomerization, and α-synuclein regulating the levels of essential PUFAs, including arachidonic acid, that modulates SNARE complex formation and endocytic activity [149,150]. Studies performed in astrocytes from an α-synuclein gene-ablated mouse revealed a significant reduction in PA uptake in a brain-specific way, resulting in a compromised brain metabolism [151]. This protein also appears to play a role in triacylglycerol (TAG) metabolism, since overexpression of the A53T α-synuclein PD mutant in a dopaminergic neuronal cell line increased TAG levels through the activation the acyl-CoA synthetase enzyme and caused LD accumulation [152]. Regarding cholesterol metabolism, studies performed on the brain and astrocytes revealed significantly elevated cholesteryl esters and cholesterol upon α-synuclein defects [153,154]. Aside from this evidence, several reports have also confirmed that this protein can interact with other lipid classes, including phospholipids and other membrane lipids (reviewed in [3]).

Genomic studies have also pointed towards a link between alterations in genes related to lipid metabolism and trafficking, as well as increased PD risk [155,156]. Mutations in the glucosylceramidase-beta gene (GBA), which encodes the lysosomal enzyme glucocerebrosidase (GCase), are a major contributing factor in the development of PD. Even though the exact mechanisms by which GCase dysfunction enhances the risk of PD have not been fully uncovered, this enzyme, which catalyzes the production of ceramide and sphingosine, has been proposed to interact with α-synuclein, promoting its aggregation and buildup [157]. Furthermore, other studies suggest that the loss of this enzyme’s activity can cause the accumulation of its substrates and interfere with α-synuclein clearance [158]. A recent study suggested modifications in the lipidomic profile of GBA KO astrocytes, with cells exhibiting higher levels of sphingomyelin, lower levels of TAGs, and overall lower uptake and storage of FA analogs in LDs [159]. Defects in the calcium-independent phospholipase A2 gene (PLA2G6), which catalyzes the hydrolysis of glycerophospholipids to produce FAs, are linked with parkinsonism and have been found to induce mitochondrial dysfunction, increase lipid peroxidation and cause neurodegeneration in Drosophila models and PD patient-derived fibroblasts [160]. Moreover, during ER stress, PLA2G6 was shown to be activated and promote sphingomyelin hydrolysis and ceramide accumulation [161], further strengthening the link between this enzyme’s impaired function and PD risk. Reports have shown that downregulation of SREBF1 (the gene encoding for the transcriptional factor SREBP1 [162]) reduces the expression of Niemann-Pick disease type C protein 1 (NPC1) genes, which are responsible for the transport of cholesterol, causing its accumulation in lysosomes and late endosomes [163]. Aside from this role, SREBF1 is also involved in the coordination of PINK1/Parkin-mediated mitophagy [164], with SREBF1 KO blocking Parkin translocation to the mitochondria and affecting PINK1 stabilization in the OMM. The mitophagy defects were rescued upon supplementation with exogenous cholesterol and FAs, with a slight improvement in PINK1 stabilization. These studies suggest that lipid synthesis might play a role in overall mitochondrial homeostasis, with alterations in these pathways potentially contributing to PD pathophysiology. Alterations in other lipid-related genes, including Lysosomal integral membrane protein 2 (SCARB2/LIMP2) and Diacylglycerol kinase theta (DGKQ), have also been suggested to increase susceptibility to PD (reviewed in [3]).

Finally, building evidence has linked PD pathogenesis with oxidative stress and inflammation. Increased oxidative stress is known to counterbalance the antioxidant mechanisms of cells, leading to cytotoxicity and degeneration [165]. After release, ROS can target PUFAs in membrane glycerophospholipids, enhancing the lipid peroxidation reaction and causing membrane dysfunction, leakage and cell death. Additionally, markers of oxidative damage and lipid peroxidation have also been found elevated in PD patients [166].

More recent research has uncovered metabolomic defects in PD models and patient samples, suggesting FAs as promising diagnosis markers for this disease [167,168]. 6-OHDA-induced PD models show significant decreases in mean chain length and saturation of FAs, which were also observed upon treatment with the mitochondrial pyruvate carrier inhibitor MSDC-0160 [169]. Interestingly, treatment with this drug was able to increase the expression of CPT1 and the formation of ketone bodies, suggesting an increase in FAO. Changes in FA metabolism were also reported in other mouse and rat models of PD [170,171]. Human studies of PD have reported decreased levels of long-chain acylcarnitines and increased levels of LCFAs, suggesting that impaired β-oxidation might be an early event during disease progression [168]. Later, by treating PD patients with Zonisamide, the defective FA intermediate levels were rescued and an improvement of the patient’s tremor scores was observed [172]. Complementing this data are multiple studies that have found alterations in FA metabolism profiles when comparing PD patients with healthy controls [173,174,175,176].

Collectively, these findings support the view that lipid metabolism dysregulation in PD cannot be interpreted solely as either a primary cause or a downstream consequence of pathology, but rather as part of a dynamic and reciprocal process that both shapes and is shaped by disease progression. This bidirectional relationship positions lipid alterations as critical modulators of neuronal vulnerability, acting to amplify the known PD pathological cascades. Although a deeper understanding of these mechanisms is needed to disentangle these very complex processes, targeting lipid pathways may offer a potential therapeutic strategy to tackle PD pathological cascades, ultimately resulting in neuronal degeneration. Importantly, given the central role of lipids in cellular energetics and organelle function, these disturbances are likely to have profound implications for mitochondrial homeostasis, providing a mechanistic link to the mitochondrial dysfunction that characterizes PD and setting the stage for further exploration of lipid–mitochondria interplay.

## 6. Impact of Lipid Alterations in Mitochondrial Function

Mitochondrial homeostasis is critically dependent on lipid metabolism, which influences multiple interconnected aspects of the organelle’s biology. Alterations in lipid balance can affect mitochondrial function through several key axes, including the regulation of membrane structure, the control of bioenergetic activity, the maintenance of redox balance, the modulation of mitochondrial dynamics and quality control, and the coordination of apoptotic signaling pathways.

### 6.1. Changes in Mitochondrial Membrane Structure and Cristae Morphology

Alterations in phospholipid composition, particularly in CL, PC and PE, play a central role in regulating the mitochondrial membrane structure, and their imbalance can compromise the organelle’s morphology or stability. A proper phospholipid homeostasis is therefore essential to confer functional and structural specificities to mitochondria, and ultimately to sustain their bonafide function.

PC is an essential component of the mitochondrial membrane, regulating the assembly of the OMM translocase TOM and the biogenesis of β-barrel proteins [177,178], and deficient production of phosphocholine and PC cause impairments in the mitochondrial structure and function. CL accounts for approximately 15–20% of the total mitochondrial phospholipids and presents a unique structure that confers IMM its characteristic curvature-sensing abilities [179,180]. This phospholipid is therefore critical to maintaining cristae morphology and stability [181]. CL remodeling, the process in which this phospholipid matures and acquires specific highly unsaturated fatty acyl chains, is essential to sustaining the multiple CL functions in the mitochondria. Mutants of defective remodeling of CL have been reported to exhibit structurally abnormal organelles, particularly enlarged mitochondria with hyperdense cristae [182,183]. Aside from being a structural component of the IMM, CL plays also a central role in energy production, quality control and dynamics, and mediation of the apoptosis process [184,185].

The formation of mitochondrial cristae also relies on the correct supply of proteins and phospholipids to the IMM. Studies in yeast revealed that loss of the Mitochondrial distribution and morphology protein 35 (Mdm35) a protein involved in the import/stabilization of Ups proteins, impacted the mitochondrial cristae structure [186]. Such functional complexes are critical for the transfer of PS from the OMM to the IMM for the synthesis of PE, pointing towards a potential role of the intramitochondrial phospholipid transport in cristae formation.

### 6.2. Changes in Mitochondrial Bioenergetics and OXPHOS Complexes

The phospholipids PE and CL are also critical for the maintenance of the mitochondrial respiratory functions [187,188]. CL is known to bind and help stabilize the mitochondrial complexes in the IMM, including the OXPHOS complexes and translocator proteins such as the TIM23 complex [189,190]. Moreover, this phospholipid is also required for the assembly/stabilization of supercomplexes, with studies documenting that CL depletion causes supercomplex dissociation, which can be restored upon repletion or decreased lipid saturation [191,192]. The impact of decreased CL levels in the stability/activity of the respiratory supercomplexes consisting of complexes II and IV has also been observed [193,194]. Likewise, CL-deficient mitochondria possess dissociated dimers of ADP/ATP carrier and other ADP/ATP carrier-related complexes [195]. Cryoelectron tomography studies performed in flight muscle of wild-type and CL-synthase mutant flies observed a structural disorganization of complex V, suggesting that CL is critical for both the oligomerization and assembly of ATP synthase complexes, consequently affecting energetic efficiency in these models [196].

A balance between PC and PE is also essential for the regulation of energy metabolism and mitochondrial function. Inhibiting Phosphatidylethanolamine N-methyltransferase (PEMT), the enzyme that converts PE into PC, in mouse 3t3-L1 mature adipocytes or mutating PEMT in mouse models result in enhanced coenzyme Q levels [197], which increases the respiratory chain electron transport and ATP production [198]. To support this, evidence has demonstrated that specific mutations in the choline kinase enzyme, involved in the synthesis of phosphocholine, an intermediate in the PC metabolism, results in impaired mitochondrial respiration and ATP production, and results in increased ROS production and mitophagy induction in mutant mice models [199].

Lipids also appear to control upstream bioenergetic pathways and energy metabolism in mitochondria. A study revealed a decreased carbon flux from glucose to acetyl-CoA, which was due to a decreased activity of the pyruvate dehydrogenase enzyme, in a CL-deficient mouse cell line, supporting the role of CL in the overall TCA cycle regulation and an axis between lipids and the general mitochondrial energy metabolism [200]. Moreover, the activity of the pyruvate carrier protein is enhanced in the presence of CL compared with other phospholipids [201]. Regarding lipid metabolism pathways, a study performed on yeast revealed that cells expressing a hyperactive mutant form of the small GTPase ADP-ribosylation factor 1 (Arf1) had low levels of FA transporters and β-oxidation enzymes, which resulted in an increased accumulation of FAs in LDs and a further decrease in ATP levels [202]. Adding to this, these models presented high levels of mitochondrial fragmentation, suggesting that lipid metabolic pathways can directly control mitochondrial network architecture. In a different study, authors have shown in a mouse model of PE deficiency that the formation of FAs and triglycerides was increased, leading to obesity and liver steatosis, and that these phenotypes were restored upon choline supplementation via enhanced lipolysis and PPARα-activated FAO [203]. Lastly, CL is known as a regulator of the activity of the acylcarnitine translocator CACT enzyme, essential for the earlier steps of the FA degradation pathway [204].

Defects in the degradation of FAs are also known to cause mitochondrial dysfunction and are involved in the pathophysiology of multiple FA-related disorders. Accumulation of FAs and FA intermediates are toxic to mitochondria and can inhibit the respiratory chain complexes and cause accumulation of ROS [205,206]. The impact of modifications in the FAO pathways on the mitochondrial bioenergetics has been extensively reviewed in [207].

### 6.3. Changes in ROS, Redox Balance and Apoptotic Signaling

The mitochondrial redox balance consists of the crucial equilibrium between the production of ROS derived from the bioenergetic processes occurring in the organelle, and their removal by antioxidant systems. A disruption of the redox balance, where ROS accumulation surpasses the antioxidant capacity of cells, induces significant mitochondrial dysfunction, including excessive oxidative stress, mitochondrial DNA damage and impaired energy production [208,209].

Imbalances between FAS and FAO processes in mitochondria are tightly regulated by shifts in metabolism, including control of β-oxidation rates, induction of TCA cycling and regulation of OXPHOS. However, all these mechanisms generate ROS and accelerate their accumulation, promoting cellular inflammation and apoptosis [210,211]. High ROS accumulation is a known inducer of lipid peroxidation, the process of oxidative degradation of lipids. Lipid peroxidation can impair the mitochondrial redox balance by targeting multiple mitochondrial pathways. Mitochondrial phospholipids, especially CL, are highly prone to oxidation, and studies have suggested that upon increased ROS accumulation, the production of reactive lipid species occurs, which affects mitochondrial redox signaling and activates apoptosis [212]. ROS-induced oxidative CL damage can also impact mitochondrial OXPHOS activity, leading to loss of complex I function and impaired electron transport, which can be restored upon restoration of CL levels [213]. Furthermore, the mitochondrial permeability transition pore (MPTP) opening and high permeability in response to increased calcium levels and oxidant stress is also regulated by CL oxidation [214].

The localization of CL within the mitochondria can also act as a marker of apoptosis. Under conditions of high oxygen stress and damage, the exchange of CL positioning from the IMM to the OMM (a process called CL remodeling) causes the formation of pro-apoptotic factors that result in mitochondrial membrane leakage and cytochrome C release to the cytosol [215,216]. For a more detailed overview of the role of lipids in apoptosis and redox balance, see review [217].

### 6.4. Changes in Mitochondrial Dynamics and Quality Control

Mitochondrial fusion and fission processes are continually regulated by several mechanisms to optimize the different mitochondrial functions. Alterations in the levels of different mitochondrial phospholipids can impact these dynamic mechanisms, and direct interactions have been reported between lipids and fusion/fission machinery proteins.

Studies on yeast revealed that loss of CL and PE result in increased mitochondrial fragmentation, loss of mitochondrial DNA and lower mitochondrial membrane potential, characteristics of alterations in the fusion process [218]. This was further validated by deleting the Dynamin-1 (DNM1) protein, essential for the fission process, which caused increased mitochondrial fusion. CL also appears to directly regulate the mitochondrial fission process through a direct interaction with Drp1, promoting its GTPase activity and oligomerization [219]. The externalization of CL to the OMM mediated by oxidative stress can further promote Drp1 stability and activate fission, supporting a crucial role for CL in sustaining an optimal mitochondrial network [220]. Adding to this role, studies have also suggested that CL is essential for IMM fusion pore formation, a process that depends on CL binding to Opa1, therefore regulating mitochondrial fusion [221]. Finally, FAO and mitochondrial dynamics appear to be interdependent processes, with studies suggesting mitochondrial fragmentation as a regulator of FA degradation via CPT1 activation [222], and CPT2-deficient models having compromised mitochondrial biogenesis, and structural/morphological mitochondrial defects [223].

Lipids have also been pointed out as regulators of other quality control mechanisms, including mitophagy. CL has been reported to interact with different mitophagy-related proteins, including LC3 and Beclin 1 [224,225], assisting in the elimination of defective organelles from cells. FAO-deficient CPT2-null mouse models revealed a suppression of mitophagy, with a particular impairment of PINK1/Parkin signaling [226]. Other evidence has also demonstrated that SFA supplementation in the form of PA increases the stability of the mitophagy receptor FUNDC1 and directly impacts on mitochondrial quality control [227]. The impact of FAs in the regulation of this mitochondrial elimination process has been further demonstrated by the observation that high-fat diet-fed mice present increased autophagosome formation and higher activation of autophagy receptors compared with low-fat diet-fed animals [228]. In the same study, authors observed that palmitate supplementation of cultured myoblasts provoked a decrease in mitochondrial membrane potential and lead to accumulation of upstream mitophagy markers, supporting a role for lipids in monitoring the upstream mitochondrial autophagy pathways.

To conclude, all the above evidence seems to point out that mitochondrial homeostasis and function critically depend on lipid composition and metabolism. Alterations in mitochondrial lipids, especially CL, PE and PC, and accumulation of FAs can disrupt the organelle’s membrane architecture, impair the activity of the respiratory chain complexes and destabilize cristae structure. These lipid changes can also compromise the mitochondrial OXPHOS activity, increase the accumulation of ROS and oxidative damage, and promote apoptotic signaling.

## 7. PINK1 as a Regulator of Lipid Metabolism and Neuronal Homeostasis

Lipid dysregulation interferes with mitochondrial dynamics, protein import and mitophagy, ultimately leading to global mitochondrial dysfunction. In this context, particular attention has been drawn to the role of PINK1, a key regulator of mitochondrial homeostasis as discussed in Section 2. The relevance of a balanced lipid homeostasis in the brain and the impact of alterations in those pathways for PD have been further supported by recent reports on a role for PINK1 in the metabolism of lipids.

A study performed in isolated primary mouse adipocytes has unveiled that PINK1 downregulation decreases the expression of the FA transport gene Fabp4, whose activity is controlled by the levels of intracellular FAs via the PPAR [229]. In the same study, several single-nucleotide polymorphisms (SNPs) in or near the PINK1 locus were significantly associated with differences in plasma-free FA concentrations in European-ancestry individuals, suggesting that PINK1 influences systemic lipid metabolism in humans.

Evidence has shown that, in diabetic mice and AD patients brain tissue, the expression of PGC-1α is decreased [230]. This transcription co-factor is a key regulator of mitochondrial oxidative function and metabolic homeostasis, with several studies pointing towards a role in the FAO [231,232,233]. In this study, the decreased PGC-1α levels negatively impacted the size/number of LDs and the mitochondrial bioenergetic capacity to use FAs as fuel. Interestingly, it was further revealed that PINK1 (whose expression levels were decreased in the same samples) was required to potentiate the activity of PGC-1α, rescuing the previously observed defects in lipid metabolism. The authors suggest that PINK1 might be targeting PGC-1α via phosphorylation, and that when absent, it impacts the stability and/or function of this transcription factor, making it inept in metabolizing lipids.

Mass spectrometry and proteomics analysis of isolated mitochondria obtained from PINK1 KO mice showed modifications in the FA metabolic pathways, particularly through decreased levels of critical β-oxidation enzymes including ACADs, ACAA2 and ETFB [234,235]. When comparing the levels of these proteins in young vs. older animals, a similar protein profile was detected, suggesting that PINK1 partakes in these metabolic processes from a very early stage. Functional proteomics revealed alterations in different mitochondrial metabolites/substrates and key proteins involved in mitochondrial bioenergetics, while functional assays pointed towards reduced mitochondrial OXPHOS efficiency and increased proton leakage [235]. These reports support the notion that the lipid metabolic pathways are disrupted in PINK1 KO mouse brains, which could contribute to the compromised mitochondrial respiratory efficiency and energy homeostasis characteristic of this disease. Similarly, observations from a proteomics study conducted in the synaptic mitochondrial pool of PINK1 KO rat striatum identified protein expression changes in lipid metabolism proteins, including downregulation of FAO (such as ACADL) and electron transport carrier proteins (ETFA and ETFB) [236]. This was accompanied by severe defects in the OXPHOS chain, particularly a reduction in the complex I-driven respiration, in line with previous findings [6]. This suggests that loss of PINK1 not only causes a dysfunction in the ETC, given its role in the regulation of complex I activity [32,237], but also in the lipid oxidation pathways that feed into that chain, exacerbating the mitochondrial respiratory chain dysfunction.

Modulation of lipid metabolism was also found to supress PINK1-related mitochondrial defects. A study performed in PINK1 mutant flies revealed that dietary supplementation with C18:0 FA improves the ATP levels and behavioral phenotypes observed in these models. Interestingly, feeding with this FA also rescues mitochondrial fragmentation observed in Parkin loss-of-function flies [138]. This is hypothesized to occur through the activation of a transcriptional mechanism mediated by TFR1, whose function is regulated by C18:0, and will trigger a multi-player signaling cascade, ultimately improving mitochondrial activity and dynamics. Genetic and pharmacological inhibition of the FA synthase (FASN) in various PINK1-null models, including mouse embryonic fibroblasts and PD-patient derived cells, was found to decrease PA levels and enhance the levels of CL [8]. Directly adding CL to isolated mitochondria from these models caused a rescue of the defective complex I activity and improved the electron flow between this complex and ubiquinone. This reveals that this phospholipid can functionally compensate for the PINK1 loss of function and points towards a possible lipid-modulation mechanism of the IMM to ameliorate PD-related mitochondrial impairments.

So far, although the mechanisms by which PINK1 might be involved in lipid metabolism regulation have not been fully uncovered, it is reasonable to hypothesize a phosphorylation-mediated control, considering its role as a mitochondrial kinase. To support this claim, it was observed that mimicking the phosphorylation of the medium-chain ACAD (MCAD) in PINK1 KO Drosophila flies is able to rescue the behavior defects characteristic of this model [9]. Furthermore, analysis of the metabolic profile of these PINK1-null flies revealed serious disruptions in multiple metabolites, including acylcarnitines, that were partially restored upon introduction of the MCAD phosphomimetic.

Studies have demonstrated that FAO is negatively regulated by increased ceramide levels [238]. In PINK1-null flies and patient-derived fibroblasts, which present high levels of this lipid species, β-oxidation was significantly impaired [10]. This effect was rescued upon inhibiting ceramide synthesis, but not when the entry of FAs was prevented in these models through pharmacological or KO approaches. Interestingly, carnitine supplementation not only restored β-oxidation levels but also improved the mitochondrial respiratory capacity and behavioral defects in flies, pointing towards a protective role for lipid metabolism in ETC dysfunction.

Considering the role of FAs as bioactive signaling molecules, evidence has also shown that FAs can play a role in the regulation of PINK1’s expression and/or function in cells. It was demonstrated that PA modulates PINK1 transcription through the increase in acetyl-CoA levels, which drives acetylation of the transcription factor Forkhead box protein O3 (FOXO3a) [239]. The decrease in PINK1 messenger RNA expression was linked with an impaired mitophagy process, increased mitochondrial DNA release and inflammasome activation, processes that were rescued upon pharmacological inhibition of FOXO3a acetylation, suggesting a link between lipid overload and poor mitochondrial quality control. Although this study was conducted in isolated macrophages from diabetic mice and wild-type mice conditioned with palmitate to induce metabolic stress, it is possible that such processes also occur in the brain, especially considering the role of microglia in neuroinflammation. Microglia are considered to be the brain’s resident immune cells, and their activation/function has been shown to be affected by lipids [240]. Moreover, evidence has shown that these cells accumulate LDs during aging, a process that contributes to neuroinflammation and can have implications in neurodegenerative diseases such as PD [241]. By contrast, a more recent study reported that PA can activate PINK1 expression and that PINK1 knockdown enhances PA-induced inflammation in adipocytes. This caused an impairment in mitochondrial function, evoking the release of mitochondrial DNA and activation of the cyclic GMP-AMP synthase (cGAS)- stimulator of interferon genes (STING) cascade, triggering inflammation [242]. Another report revealed that, in adenine-induced uremic rats, omega-3 FA administration can reverse the downregulation of different mitochondrial proteins, including PINK1, in both heart and kidney tissues [230]. This helped recover the PINK1-mediated mitophagy, showcasing the importance of this process for the clearance of damaged mitochondria in these tissues. Interestingly, the expression of BCL2 interacting protein 3 (BNIP3) and NIX, two other mitophagy regulators, was inversely regulated in the kidney, with FA administration causing decreases in the expression of these proteins. The results point to a differential mitophagy regulation across these tissues and underscore the multifaceted role of FAs in mitochondrial homeostasis. More recently, an inverse correlation has been made between PINK1 gene expression and intramuscular triglycerides deposition in pigs [243]. Loss of PINK1 further impaired mitochondrial respiration, increased mitochondrial biogenesis, and perturbed mitochondria–ER contact sites. This suggests that PINK1 function and inter-organelle interactions are indispensable for lipid deposition. These findings illustrate that FAs do not exert a uniform effect on PINK1 and that instead, their impact appears to be highly context dependent. Differences in cell type, metabolic state, duration of lipid exposure or concentrations, and inflammatory environment are likely to induce differential roles for FAs as expression regulators, ultimately determining whether PINK1 activation serves a protective mitochondrial quality control function or, conversely, becomes suppressed, thereby promoting mitochondrial dysfunction.

Taken together, these studies point to an emerging and multifaceted interplay between PINK1 and lipid metabolism, highlighting lipid homeostasis as a central component of mitochondrial health in the context of PD. Beyond its already well-established functions, PINK1 appears to participate in the regulation of FA uptake, β-oxidation and lipid-dependent signaling pathways that support mitochondrial activity. In parallel, accumulating evidence demonstrates that alterations in lipid availability, composition or metabolic stress can modulate this kinase’s expression and activity, thereby influencing mitochondrial quality control and inflammatory signaling. Importantly, these interactions are highly context-dependent, varying with cell type, metabolic state, lipid species, and duration of exposure. While the precise molecular mechanisms linking PINK1 to lipid metabolic pathways are not yet fully understood, the convergence of genetic, proteomic, and metabolic data underscores a tight coupling between lipid metabolism and mitochondrial dysfunction in PD. Elucidating how disruptions in this lipid–PINK1 axis contribute to neuronal vulnerability may open new avenues for therapeutic strategies aimed at restoring metabolic balance, mitigating mitochondrial dysfunction, and ultimately delaying disease progression.

## 8. Future Perspectives and Therapeutic Outlook

Mitochondria are very dynamic organelles that coordinate a plethora of critical functions in the cell, ranging from energy production and calcium homeostasis to ROS regulation and apoptosis management. These organelles are also complex metabolic centres that can synthesize and degrade multiple molecules and substrates, including amino acids, proteins and lipids, to sustain cellular activities. Lipids are a major class of biomolecules whose importance has been well-documented in cells. They are integral parts of both the cellular and mitochondrial membranes and can be used as fuels for energy production. Lipids can also mediate signaling cascades and are essential regulators of several mitochondrial functions. It is because of these critical roles that lipids require a tight regulation to ensure the proper functioning of the cell. Within the different cell populations in mammalian systems, neurons are particularly susceptible to lipid alterations, considering that they rely on these molecules for their growth, maintenance and survival. Given their importance, it is not surprising that defects in lipid-related pathways are at the root of multiple metabolic and neurodegenerative disorders, including PD.

**Figure 3:**
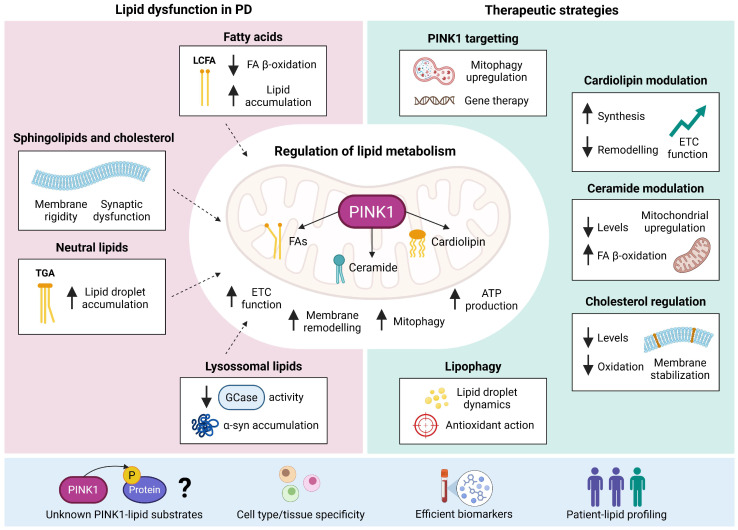
PINK1 as a central regulator of lipid–mitochondrial crosstalk in PD. (**Left**) Lipid homeostasis is impaired in PD, with defects in β-oxidation causing accumulation of FAs and resulting in metabolic imbalance. Altered sphingolipid and cholesterol composition/interactions affect membrane properties and disrupt synaptic function in neurons, compromising the normal brain activity. Excessive neutral lipid storage, including TAGs in lipid droplets, in cells induces lipotoxicity and precedes neuronal degeneration. GBA mutations, commonly found in PD patients, affect GCase enzymatic activity and hinder lysosomal lipid metabolism, compromising α-synuclein processing. These lipid metabolic defects exacerbate mitochondrial dysfunction, promote neuroinflammation and compromise neuronal viability. (**Middle**) PINK1 coordinates multiple mitochondrial lipid handling pathways via interaction with cardiolipin, regulation of ceramide balance and modulation of FA utilization. This places PINK1 as a central hub for the regulation of mitophagy, membrane dynamics, electron transport chain activity and ATP production. (**Right**) Emerging therapeutic strategies targeting PINK1–lipid crosstalk, including activation of PINK1-mediated functions that are upstream of the lipid metabolic defects. Approaches targeting the modulation of the affected lipid species can prove efficient in rescuing mitochondrial functions, including OXPHOS capacity, mitophagy and membrane characteristics. Enhancement of lipophagy pathways via stimulation of FA degradation or direct treatment with FAs can restore lipid homeostasis and is neuroprotective. (**Bottom**) Key future directions in PD therapeutics highlight unresolved questions, including identification of PINK1-dependent lipid substrates to better target the affected pathways. The metabolic specificities of the different cell types and tissues, particularly those of the brain, should be considered when developing lipid-targeted therapies. Development of robust biomarkers to track a patient’s treatment progress, as well as stratification according to the lipidomic/metabolomic profiles, should be considered for precision therapies in PD. FA: Fatty acid; LCFA: Long-chain FA; TGA: Triacylglycerol; GBA: Glucosylceramidase-beta; GCase: Glucocerebrosidase; α-syn: α-synuclein; ETC: Electron transport chain; OXPHOS: Oxidative phosphorylation chain; ATP: Adenosine triphosphate; PINK1: PTEN-induced putative kinase 1; PD: Parkinson’s disease. Arrow notation: Bold arrows indicate increased and decreased processes, respectively. Thin arrows indicate the direction of biological processes or conceptual/functional relationships between components and should not be interpreted as changes in activity.Created in BioRender. Ramos, A. B.; Morais V.A. (2026) https://BioRender.com/c8gyem5 (accessed on 7 April 2026).

Although the emerging literature places lipid metabolism close to the core of PD, some questions remain to be clarified to better comprehend which lipid changes are causal, which are compensatory and which are more easily adaptable for therapy options (Figure 3).

As reviewed above, many studies on PD models show lipid homeostasis defects accompanied by mitochondrial dysfunction and neuronal degeneration processes. However, the temporal order in which these processes occur remains unclear, hindering our understanding of the disease’s pathways. Concerning PINK1’s role in these processes, studies have hinted towards distinct and potentially competing mechanisms, with lipid accumulation triggering mitochondrial dysfunction in some PINK1-deficient models, or lipids ameliorating functional phenotypes evoked by a PINK1 defect in others [8,10]. Such conundrum suggests that lipid modifications can both exert protective or detrimental effects depending on the cellular context or disease stage.

The fact that each of the described lipid species are compartment-specific and have distinct roles in cells and in mitochondria can also harm our understanding of the disease’s pathophysiology. Although CL is an IMM-specific lipid, other mitochondrial phospholipids such as PE and PC are also present in other organelles, including the ER, Golgi apparatus and in cellular membranes. Ceramides are mainly present in the mitochondria and in lysosomes, and cholesterol is mostly present in transport membranes, with higher concentrations in synaptic vesicles [244,245]. Due to these broad cellular and tissue distributions, it is unlikely that such lipids play interchangeable roles, raising the need for spatial lipidomics in defined neuronal compartments and glial populations to better comprehend how they are affected in the context of PD.

Even though PINK1 is classically framed as having a role in sensing mitochondrial damage and mediating Parkin-mediated mitophagy, the literature now points towards much broader and critical roles of this kinase, from the regulation of ER–mitochondria contact sites to metabolic rewiring, mitochondrial structural regulation and dopamine handling in neurons [246,247,248]. More recently, studies have implied an involvement of PINK1 in lipid homeostasis, adding a further layer of complexity to this mitochondrial kinase. However, it remains unresolved whether this function results from a direct output of PINK1-mediated phosphorylation, or if they are secondary consequences to other PINK1 functions in cells. Finding specific phosphorylation substrates involved in lipid metabolism pathways would therefore prove useful to determine whether PINK1-targeted therapies can normalize lipid homeostasis in PD models.

Recent work on PD consistently points that PINK1-related forms of the disease are not isolated from the broader PD genetics. As previously described in Section 2 of this review, multiple PD-related genes can cause hereditary forms of this disorder, but several genetic alterations can also contribute to the development of late-onset sporadic PD forms. In line with this, other PD-related genes aside from PINK1 have also been shown to influence lipid metabolism. For example, SREBF1 connects lipogenesis to PINK1-mediated mitophagy, linking PD-familiar pathways to processes also implicated in sporadic PD [164]. Parkin regulates neuronal lipid homeostasis through SREBP2 and lipoprotein lipase, expanding its role from a mitochondrial quality control protein to a broader metabolic regulator [249]. Finally, GBA1 mutations, commonly found in PD patients, highlight that multiple lipid disturbances co-occur with autophagy defects and α-synuclein deposition [250]. All these and other evidence point to different PD subtypes converging on a shared biologically pathway affecting lipids, which has downstream implications to mitochondria and normal cellular functioning.

Most of the experimental work on these lipid-involving pathways still relies on cell models or on broad toxin models. However, neurons, astrocytes, microglia and other brain cell populations differ sharply in their lipid utilization and mitochondrial demand [251,252]. Moreover, evidence suggests that different neuronal subtypes, especially dopaminergic neurons, may also have divergent lipid metabolisms [248], which greatly increases the need to use patient-derived neurons, organoids and other in vivo models to perform these lipid-targeted studies and better tackle the PD pathology mechanisms.

Lastly, the progress from basic discoveries to effective therapies remains slow in PD, despite the growing knowledge about lipid involvement in the disease pathways. This occurs mainly due to the lack of reliable biomarkers to measure the impact of the modulation of these mechanisms in living patients, making it difficult to confirm treatment efficiency. Although some patient stratification has been made possible by using patients’ serum, sebum and CSF samples, as well as by adopting multi-omics approaches, the field still lacks validated biomarkers that can target specific disease-related mechanisms or can predict treatment responsiveness [136,253,254].

The therapeutic landscape in PD is becoming progressively more tangible. Rather than focusing on a single lipid target, evidence supports a multitargeted approach, with overlapping strategies that aim to restore mitochondrial membrane quality, reduce lipotoxic stress, improve lysosomal sphingolipid handling, and reactivate PINK1-dependent quality control.

Targeting the PINK1-mediated pathways remains one of the more appealing approaches given that it aims to rescue mitophagy and other mitochondrial-related functions upstream of the lipid defects observed in PD. Niclosamide analogs can activate the PINK1 kinase activity in cells and primary neurons, inducing Parkin recruitment and mitophagy [255]. Novel PINK1 activators have also been identified and observed to ameliorate PD pathology, including α-synuclein deposition and mitophagy defects, in animal disease models [256,257]. Such studies provide the first steps towards developing disease-modifying strategies, even though their translation to efficient therapies will require an improved specificity and the development of robust biomarkers to assess pathway engagement in patients.

A different approach would be to target CL and mitochondrial membrane remodeling. As mentioned in the earlier sections of this review, CL is an integrant phospholipid of the IMM with a central role in sustaining the respiratory chain function, membrane organization and mitophagy. Under PINK1 deficiency, increasing CL levels can rescue electron transfer defects [8]. Furthermore, studies have demonstrated that inhibition of the pathological CL remodeler Acyl-CoA:lysocardiolipin acyltransferase-1 (ALCAT1) appears to be protective in MPTP models [258], and that new compounds such as ginsenoside Rg3 and tenofovir disoproxil fumarate have shown preclinical benefits by promoting CL synthesis and restoring mitochondrial homeostasis in disease models [259]. These findings make CL one of the most promising lipids to target in the field, although its delivery to the mitochondria in the human brain, as well as its long-term effects on membrane remodeling, require further investigation.

Ceramides and other related sphingolipids can also be modulated as a therapeutic approach to PD. In PINK1 deficiency, ceramide accumulation contributes to a harmful cycle in which mitophagy impairment, decreased FAO and respiratory dysfunction act in a synergistic way, progressively increasing mitochondrial dysfunction. Concomitantly, lowering ceramide levels or stimulating FA degradation can rescue the PINK1-null phenotype [10]. In parallel, research focused on the PD-related gene GBA has outlined a broader therapeutic strategy in which restoring GCase activity, intracellular trafficking or substrate balance helps reduce α-synuclein pathology and improve lysosomal function [260]. For this family of lipids, the question of whether future therapies should target the total sphingolipid alterations or focus on specific lipid species and particular patient subgroups remains.

Other potential strategies focus on LDs, lipophagy and metabolic rewiring. Linoleic acid treatment has shown neuroprotective and anti-inflammatory effects in cellular and mouse PD models, in part due to improvement of LD dynamics and lipophagy, resulting in an antioxidant effect in these models [261]. The compound Cyclodextrin is also of interest in this context, since 2-Hydroxypropyl-β Cyclodextrin (HP-β-CD) was able to improve the PINK1-evoked phenotypes in mutant patient-derived organoids [262]. Related metabolic evidence suggests that interventions aimed at FA degradation may also be beneficial, although this area is not yet fully resolved. For example, in PINK1 deficiency, stimulation of FAO can be protective [10], whereas in a Parkin-mutant model, inhibition of the FA transporter CPT1 improves behavior and metabolic abnormalities [263]. This suggests that metabolic interventions, especially targeting FAs, may depend on different factors, including disease stage or the specific form of lipid stress present.

Lastly, cholesterol homeostasis is another potential therapeutic candidate. PINK1 loss has been linked with increased cholesterol levels, enhanced membrane rigidity and synaptic dysfunction, with some of these defects being reversed upon treatment with the cholesterol depleting drug β-cyclodextrin [248]. More broadly, cholesterol-targeting strategies have been proposed for the treatment of PD due to the evidence suggesting that an imbalance between cholesterol and its oxidized derivative oxysterol may affect α-synuclein trafficking and membrane behavior [264]. However, as cholesterol metabolism is tightly regulated in the brain, any therapeutic interventions must be carefully designed to avoid wider systemic effects.

Taken together, two therapeutic strategies emerge as particularly promising. The first targets PINK1 function directly, as its regulated pathways act near an upstream point that may secondarily normalize several downstream lipid defects. The second involves direct lipid remodeling, with multiple strategies already demonstrating biochemical and phenotypic rescue in various PD-relevant systems. Regarding the former, further experimental evidence is needed to strengthen the link between PINK1 and its role in modulating lipid metabolism, including the identification of bonafide phosphorylation substrates and a clearer understanding of the involved pathways. In the case of the latter, lipid-targeted therapies should not be viewed as a single, “one-size-fits-all” solution, particularly because the same lipid pathway can be beneficial in one cellular context but detrimental in another. Consequently, modulation of lipid content or function may produce different outcomes depending on genotype, cell type or disease stage. The optimal strategy is therefore a precision approach in which lipid-targeted treatments are tailored to the patient’s specific biochemical state, and the restoration of PINK1-related pathways is pursued concurrently with the direct lipid restructuring, offering a framework for precision-targeted therapies that could simultaneously restore mitochondrial function and correct lipid dysregulation in PD.

## 9. Translational Challenges in Parkinson’s Disease: Lessons from Clinical Trials

Even though many therapeutic strategies have shown great promise in preclinical trials, no therapy so far has conclusively demonstrated neuroprotective or disease-modifying effects in PD. In vitro and in vivo data have identified robust compounds targeting major PD hallmarks, such as oxidative stress, mitochondrial dysfunction, neuroinflammation and protein aggregation. However, despite their preclinical potential, these neuroprotective drugs have failed to show efficacy in clinical trials [265,266,267]. The existing gap between promising preclinical data and failure to translate the results into effective therapeutic strategies remains one of the major obstacles in PD research and should be continuously reviewed and updated in light of emerging evidence and insights in the field.

Many reasons could be at the root of this mismatch, including the fact that most widely used preclinical models do not reproduce the complete physiology or progression of human PD. Acute toxin models such as MPTP and 6-OHDA induce rapid and often extensive dopaminergic injury, whereas human PD evolves slowly over years and implicates broader multisystem pathology [268]. Although neurotoxin-based models have been useful for probing mechanisms and screening potential therapeutic compounds, they are limited as predictors of clinical disease modification because they often lack the progressive course, multiregional degeneration and pathological features observed in human disease. Additionally, many animal studies test compounds in designs that are closer to prevention than treatment of disease. Drug administration often starts prior or simultaneously as the toxin exposure, whereas in clinical trials, patients begin treatment after substantial neuronal dysfunction and loss. This design mismatch in preclinical studies can overestimate the therapeutic benefits and weaken their relevance for later-stage human trials [269].

A different perspective is the proposal that degeneration in idiopathic PD may follow a very slow single-neuron process affecting vulnerable dopaminergic neurons individually rather than the massive and synchronized injury induced by exogenous toxins [270], which helps to explain why acute toxin-based models may not predict clinical efficacy well. Simultaneously, accumulating evidence suggests that PD is not restricted to nigral dopaminergic cell loss, but instead involves widespread neuronal populations, glial dysfunction, peripheral autonomic and enteric tissues, and gut–brain axis alterations [271,272,273]. This broader view may help explain why therapies that appear neuroprotective in models focused on substantia nigra neurons often fail in patients, as they do not address the full biological complexity of the disease. This perspective is particularly relevant to lipid metabolism, since metabolic and lipid disturbances in PD extend beyond vulnerable neurons to include glial, peripheral, and systemic components [274].

A second obstacle in this translational problem is related to a late therapeutic intervention. Even if a target is biologically credible, clinical trials can fail because treatment is introduced too late. By the time motor PD symptoms are diagnosed, a substantial amount of neuronal degeneration has already occurred, narrowing the window in which neuroprotective intervention can produce measurable benefits for patients [266,275]. This timing issue is especially relevant for therapies that aim to slow down degeneration instead of producing a rapid symptomatic effect [276].

The biological heterogeneity of PD remains a challenge in improving the efficacy of neuroprotective disease targets. Clinical trials commonly group together patients with different contributing disease-related mechanisms, including mitochondrial dysfunction, autophagic defects, α-synuclein handling, inflammation and lipid dysregulation [267,277]. A treatment aimed at one mechanism can therefore appear ineffective if tested in an unselected population in which that same mechanism is central only for a subset of patients. This highlights a biomarker-driven phenotyping and patient-personalized medicine instead of all-comers trial designs.

The field of PD clinical research still lacks sufficiently sensitive tools to measure true disease modification. Standard clinical scales to quantify symptoms and measure disability remain critical, but they change slowly, are vulnerable to symptomatic confounding and may not detect modest alterations in progression over typical trial durations [266,276]. This makes it difficult to separate a genuine disease-modifying effect from symptomatic benefit or background variability.

Relating to this, the field also lacks strong biomarkers for target engagement, pharmacodynamic response and disease progression. When the clinical trial outcome is negative, it is often unclear whether the underlying hypothesis was incorrect, whether the drug failed to reach or engage its target, or even if the study population and endpoints were poorly matched to the mechanism being tested [265,278]. Recent translational roadmaps therefore emphasize proof of target engagement, biomarker-supported patient selection, and the use of more sensitive longitudinal outcomes, including digital and wearable measures where appropriate.

Taken together, these points suggest that failed human trials should not be interpreted simply as failures of neuroprotection. They also reveal recurrent problems in model choice, treatment timing, patient selection and outcome measurement that should be considered to overcome the existing gap between preclinical and clinical studies. More clinically relevant preclinical designs should test interventions after pathology is established, use longitudinal readouts and validate efficacy across complementary model classes rather than relying on a single acute toxin paradigm. In parallel, future clinical studies will likely require earlier-stage or at-risk patient populations, biologically enriched populations, biomarkers of target engagement and endpoints that are sensitive to slow disease progression [267,277,278].

These lessons are directly relevant to therapies centered on PINK1 and lipid metabolism. Interventions that target mitophagy, mitochondrial membrane remodeling, ceramide handling or other lipid-linked pathways may be effective only in patients in whom those processes are major drivers of pathology. If such therapies are tested late in the disease, in biologically mixed cohorts or with insensitive endpoints, even a real mechanistic benefit could be missed. For this reason, the development of PINK1- and lipid-directed therapies should proceed together with efforts to perform patient stratification and patient subgroups and the biomarkers that show pathways engagement in vivo.

## Data Availability

No new data were created or analyzed in this study. Data sharing is not applicable to this article.
